# Differences of clinical features and prognosis between *Mycoplasma pneumoniae* necrotizing pneumonia and non-*Mycoplasma pneumoniae* necrotizing pneumonia in children

**DOI:** 10.1186/s12879-021-06469-x

**Published:** 2021-08-10

**Authors:** Beilei Yang, Weili Zhang, Wenjing Gu, Xinxing Zhang, Meijuan Wang, Li Huang, Canhong Zhu, Yongdong Yan, Wei Ji, Huiping Ni, Zhengrong Chen

**Affiliations:** 1 Department of Respiratory Disease, Children’s Hospital of Soochow University, Suzhou, 215003 China; 2 Department of Pediatrics, Xishan People’s Hospital of Wuxi, Wuxi, 214000 China; 3 Department of Pediatrics, The Third Affiliated of Soochow University, Changzhou, 213000 China

**Keywords:** Necrotizing pneumonia, *Mycoplasma pneumoniae*, *Non- Mycoplasma pneumoniae*, Children, Clinical features

## Abstract

**Background:**

In the past few years, *Mycoplasma pneumoniae* (Shi et al. Lancet 390:946–958, 2017) infection has been reported more in China. However, there are few studies on the clinical characteristics and prognosis of necrotizing pneumonia (NP) (Griffiths et al. Nature 583:615–619, 2020) caused by different pathogens.

**Methods:**

A retrospective analysis was performed, including 31 children with a clinical diagnosis of NP in the hospital from January 1, 2013 to January 31, 2020. A total of 11 children with MPNP were included in the observation group and the other 20 children with other pathogens were included in the control group. The clinical manifestations, laboratory data, imaging findings, treatments and outcomes were analyzed.

**Results:**

The proportion of dyspnea cases was significantly higher in the non-*Mycoplasma pneumoniae* necrotizing pneumonia (N-MPNP) group than that in the *Mycoplasma pneumoniae* necrotizing pneumonia (MPNP) group (*P* = 0.02).The LDH level of all patients in the MPNP group was higher than the normal value, with a median value of 805.0 U/L, which was significantly higher than those in the N-MPNP group (414.0 [299.9–540.6] U/L; *Z* =  − 2.518; *P* = 0.012). The white blood cells (WBCs) count of the N-MPNP group was 17.8 (11.1–21.7) × 10^9^/L, which was significantly higher than that of the MPNP group (10.2 [6.3–14.1] × 10^9^/L; *P* < 0.05). The mean time of pulmonary necrosis in the MPNP group was 20.9 ± 6.9 days, which was higher than that of the N-MPNP group (16.8 ± 6.1 days; *t* = 3.101; *P* = 0.004). The incidence of pleural effusion in the N-MPNP group (19 patients, 95%) was significantly higher than that in the MPNP group (six patients, 54.55%) (*P* = 0.013). Among them, two patients received bronchoscopy lavage at a maximum four times, and the cases of plastic bronchitis were seen only in the MPNP group (3 cases; *P* = 0.037).The length of stay was 18 (10–22) days in the MPNP group and 23.5 (13.5–47) days in the N-MPNP group and no significant difference was observed between the two groups (Z =  − 1.923, *P* =  − 0.055).

**Conclusions:**

1. MP infection is the most common infection in children with NP in the Suzhou area. There is no gender and age difference between MPNP and N-MPNP, but the bacterial infection was mainly observed in the N-MPNP group.
2. Children in the N-MPNP group have more severe clinical symptoms, were more prone to shortness of breath, had a longer hospital stay, and had earlier imaging manifestations of necrosis, whereas children in the MPNP group were more likely to have plastic bronchitis. The level of WBC and LDH and the nature of pleural effusion can be used to identify MPNP and N-MPNP to some extent.
3. The prognosis of MPNP was better than that of N-MPNP. There were no death cases. Pleural thickening, pulmonary fibrosis, and bronchiectasis were the most common sequelae. Compared with N-MPNP, the recovery time of lung imaging in MPNP was shorter.

## Introduction

Necrotizing pneumonia (NP), also named as cavitary pneumonia or cavitary necrosis, is a type of a rare and serious complication of community-acquired pneumonia in children [1–3]. With no clear definition at present, NP is mainly diagnosed depending on imaging features of the damage of the lung parenchyma structure, liquefaction necrosis, and cavity formation, typically accompanied by empyema and bronchopleural fistula [4]. Since the first report of NP in children in 1994 [5], there have been reports about NP in children worldwide [6], and more and more cases have been reported, which is closely associated with the improvement of clinicians’ understanding of NP and the clinical application of computed tomography [7, 8]. NP pathogens are diverse. Reports mainly focus on *Streptococcus pneumoniae* and *Staphylococcus aureus*. In the past few years, *Mycoplasma pneumoniae* [1] infection has been reported more in China [9]. However, there are few studies on the clinical characteristics and prognosis of NP caused by different pathogens. This study retrospectively analyzed and compared MPNP and non-MPNP (N-MPNP) in children. The clinical features and prognosis of pneumonia (N-MPNM) can provide a powerful basis for clinicians to identify NP early so that it can be treated in time and reduce the incidence of complications.

## Methods

### Patients

A retrospective analysis was performed, including 31 children (19 boys and 12 girls) with a clinical diagnosis of NP in the hospital from January 1, 2013 to January 31, 2020. A total of 11 children with MPNP were included in the observation group and the other 20 children were included in the control group, including 10 children with *Streptococcus pneumoniae* infection (one with *Acinetobacter baumannii* and filamentous fungi), four with *Staphylococcus aureus* infection (two with methicillin-resistant *Staphylococcus aureus* and one with *Haemophilus influenzae* and fungi), two with *Streptococcusviridans* infection, two with G-bacilli infection, and one with *Acinetobacter baumannii* combined with *Pseudomonas aeruginosa* and H5N6 infection. One patient was infected with *Sparganum mansoni*.

### Diagnostic criteria

NP was diagnosed as per the following criteria: (1) the patient had been healthy and met the diagnostic criteria of community-acquired pneumonia [10]; and (2) multiple low-density areas, cystic bubbles, and air or liquid cavities were observed on chest CT, which fused into large cavities, and enhanced chest CT showed reduced enhancement area, thin-walled cavity, and no edge enhancement [11, 12]. Exclusion criteria included the following: (1) children with cavitary pulmonary diseases such as pulmonary tuberculosis, pulmonary cyst with infection, and pulmonary abscess; (2) children with previous diseases such as hematological system, cardiovascular system, bronchial asthma, recurrent respiratory tract infection, and immune function deficiency; and (3) patients with incomplete medical records.

MPNP was diagnosed as per the following criteria: (1) The diagnosis and exclusion criteria of NP were met; (2) Two of the following diagnostic criteria were met at the same time: (a) serum-specific MP-IgM antibody was positive (> 1.1 COL); (b) the titer of MP-IgM antibody in both convalescent and acute phases increased or decreased by four times or more; (c) MP-DNA of nasopharyngeal aspiration (NPA) was positive (≥ 10^4^ copies/mL) using polymerase chain reaction fluorescence probe method, and (d) MP-DNA in bronchoalveolar lavage fluid (BALF) was positive (≥ 10^4^ copies/mL); and (3) other pathogens were not present.

N-MPNP was diagnosed according to the following criteria: (1) the diagnosis and exclusion criteria of NP were met; and (2) the bacteria were identified through pleural puncture fluid culture, blood culture, and BALF culture, mainly bacterial NP.

This study has been approved by the Ethics Committee of Children’s Hospital of Soochow University, and the informed consent from parents has been exempted.

### Data collection

We retrospectively reviewed the medical patients. According to the inclusion and exclusion criteria, children with NP were divided into MPNP and N-MPNP groups. The general information, clinical manifestations, laboratory examination, etiological results, imaging findings, bronchoscopy, and lavage, closed thoracic drainage, drug treatment, and prognosis of the two groups were analyzed and compared. Laboratory tests included the following: blood routine examination, high-sensitivity C-reactive protein (CRP), lactate dehydrogenase [13], erythrocyte sedimentation rate (ESR), fibrinogen (FIB), D dimer (DD), procalcitonin (PCT), biochemical complete set, lymphocyte subsets, blood culture, pleural puncture fluid culture, and high-throughput sequencing (next-generation sequencing). At the same time, the MP-DNA load of NPA and BALF, human rhinovirus, human metapneumovirus, human bocavirus, seven common respiratory viruses (respiratory syncytial virus, influenza A virus, influenza B virus, adenovirus, parainfluenza virus-1–3), and bacterial culture of NPA and BALF were also improved. Part of blood and BALF samples were collected and sent to Dinfectome Technology Co., Ltd. (Nanjing, China) for high-throughput sequencing of pathogenic bacteria.

### Statistical analysis

The statistical analyses were performed using SPSS software (IBM, version 21.0). Normally distributed data were reported as means ± standard deviations (SDs), and the two groups were compared using a two-sample t-test or the Mann–Whitney U test. Data with a skewed distribution were presented as median values (interquartile ranges: Q25–Q75), and the rank-sum test was used for the two groups. The statistical significance of the differences in the categorical variables was determined using the Chi-squared test and Fisher’s exact test. The statistical significance was determined at the two-tailed 0.05 level.

## Results

### Study population (Table 1)

**Table 1:** Comparison of gender, age, and seasonal distribution between the two groups

Clinical index	MPNP group (n = 11)	MPNP group (n = 20)	Z/χ2	P value
Female (n, %)	6,54.55%	14,70.00%	–	0.255^a^
Age (years)	5.0 (2.0–7.0)	3.5 (1.5–9.7)	−0.248	0.804
0–3 years	4 (36.36%)	10 (50%)	3.147	0.207
3–6 years	3 (27.27%)	1 (5%)		
6–14 years	4 (36.36%)	9 (45%)		
Spring	1 (9.09%)	4 (20%)	2.898	0.408
Summer	1 (9.09%)	5 (25%)		
Autumn	3 (27.27%)	2 (10%)		
Winter	6 (54.55%)	9 (45%)		

Data are presented as means ± SDs or medians (Q25, Q75)

^a^ Fisher’s exact test

All 31 patients were healthy and had no underlying diseases. According to the above diagnostic criteria, 11 patients were categorized into the MPNP group, including five boys and six girls, and 20 patients into the N-MPNP group, including 6 boys and 14 girls. No significant difference was observed in gender composition between the two groups. Regarding age characteristics, the median age of patients in the MPNP and N-MPNP groups was 5 and 3.5 years (*P* > 0.05). Moreover, patients were divided into young (0–3 years old), medium (3–6 years old), and old (6–14 years old) groups depending on their age. No significant difference was observed in the age groups and seasonal distribution.

### Clinical manifestations (Table 2)

**Table 2:** Clinical information of the two groups

Clinical information	MPNP group (n = 11)	N-MPNP group (n = 20)	P value
Hospitalization days	18.0 (10.0–22.0)	23.5 (13.5–47.0)	0.055^a^
Fever duration	13.0 (10.0–15.0)	11.5 (7.3–30.0)	0.868^b^
Clinical presentation (n,%)			
Chest pain	4 (36.36%)	9 (45%)	0.718
Dyspnea	5 (45.45%)	14 (70%)	0.255
Wheezing	2 (18.18%)	6(30%)	0.676
Digestive symptoms	6 (54.55%)	8(40%)	0.477
Feeding difficulty	5 (45.45%)	9 (45%)	1
Signs			
Shortness of breath	2 (10%)	14 (70%)	0.02
Cyanosis	3 (27.27%)	6(30%)	1
Lowered breath sound	6 (54.55%)	10 (50%)	0.555
Moist rales	5 (45.45%)	8 (40%)	
Dry rales	0	2 (10%)	
Extrapulmonary complications (n,%)			
Digestive system	4 (36.36%)	6 (30%)	1
Cardiovascular system	3 (27.27%)	3 (15%)	0.638
Nervous system	0	1 (5%)	1
Blood system	1 (9.09%)	1 (5%)	1
Rash and joint muscle system	2 (18.18%)	1 (5%)	0.281
Two systems	0	3 (15%)	0.535
Three systems and more	3 (27.27%)	1 (5%)	0.115

^a^*Z* = − 1.923, ^b^*Z* = − 0.166

1. Symptoms: all children had a fever. The total number of fever days in the MPNP group was 13 (10–15) days, and that in the N-MPNP group was 11.5 (7.3–30) days (*P* > 0.05). Other symptoms such as cough, chest pain, wheezing, dyspnea, feeding difficulties, and gastrointestinal symptoms were not statistically significant.
2. Signs: two and 14 patients had shortness of breath in the MPNP and N-MPNP groups, respectively. The proportion of dyspnea cases was significantly higher in the N-MPNP group than that in the MPNP group (*P* = 0.02). Moreover, the proportion of cyanosis cases in the two groups (27.27% and 30%) was not statistically significant (*P* = 1). A total of 16 patients with NP had decreased breath sounds, including six and 10 cases in the MPNP and N-MPNP groups, respectively. In addition, there were 13 cases of pulmonary moist rales, including five in the MPNP group and eight in the N-MPNP group. No significant difference was observed in the incidence of respiratory sounds and pulmonary rales between the two groups (*P* = 0.555).
3. Extrapulmonary complications: all NP children with extrapulmonary manifestations mainly involve the digestive system (abnormal liver function and hepatosplenomegaly), cardiovascular system (myocardial damage and pericardial effusion), blood system (hemolytic anemia and thrombocytopenia), nervous system (central nervous system infection, etc.), and rash and joint muscle diseases, mostly involving one system. In the N-MPNP group, three patients (15%) involved two systems and one (5%) involved three or more systems, whereas in the MPNP group, no patient involved two systems, but three patients (27.27%) involved three or more systems. No significant difference was observed in the incidence of extrapulmonary complications between the two groups. However, in the N-MPNP group, one patient with skin damage was diagnosed with Stevens-Johnson syndrome, and one involving the nervous system was diagnosed with encephalitis, all of which had serious extrapulmonary complications.

### Laboratory examination results

As shown in Tables 3 and 4, the LDH level of all patients in the MPNP group was higher than the normal value, with a median value of 805.0 U/L, which was significantly higher than those in the N-MPNP group (414.0 [299.9–540.6] U/L; Z =  − 2.518; *P* = 0.012). The white blood cells (WBCs) count of the N-MPNP group was 17.8 (11.1–21.7) × 10^9^/L, which was significantly higher than that of the MPNP group (10.2 [6.3–14.1] × 10^9^/L; *P* < 0.05). In addition, the median values of N%, CRP, PCT, FIB, DD, and ESR in the two groups were significantly higher than the normal values, particularly the DD value increased significantly; however, no statistical difference was observed between the two groups (*P* > 0.05). In addition, no statistical difference was observed in ALT, AST, IgA, IgG, IgM, and lymphocyte subsets between the two groups (*P* > 0.05).

**Table 3:** Comparisons of laboratory data

Laboratory data	MPNP M (Q25–Q75)	N-MPNP M (Q25–Q75)	Z	P-value
WBC (× 10^9^/L)	10.2 (6.3–14.1)	17.8 (11.1–21.7)	− 2.023	0.043
N%	74.6 (50.8–85.8)	74.4 (65.9–80.7)	− 0.413	0.680
CRP (mg/L)	53.3 (17.4–119.3)	34.8 (13.1–95.2)	− 0.661	0.509
ALT (U/L)	19.1 (9.3–475.6)	18.2 (11.4–63.2)	0.454	0.650
AST (U/L)	47.9 (24.4–237.0)	31.9 (19.4–125.7)	− 1.239	0.215
LDH (U/L)	805.0 (423.7–1029.5)	414.0 (299.9–540.6)	− 2.518	0.012
Fib (g/L)	4.8 (3.3–5.0)	5.2 (3.7–5.5)	− 1.904	0.274
DD (ug/L)	1740.0 (1165.0–2510.0)	1771.0 (1085.0–3687.5)	− 0.413	0.680
PCT (ng/mL)	0.5 (0.5–3.0)	1.5 (0.4–5.2)	− 0.745	0.456
ESR (mm/h)	65.0 (20.0–76.0)	52.5 (22.0–78.8)	− 0.062	0.951
IgA (g/L)	1.3 (0.7–1.6)	1.0 (0.5–1.5)	− 0.558	0.577
IgG (g/L)	9.6 (7.0–10.3)	8.1 (5.8–11.8)	− 0.206	0.836
IgM (g/L)	1.4 (1.0–1.7)	1.0 (0.8–1.6)	− 1.115	0.265
CD3+	57.8 (51.5–67.7)	61.1 (48.4–71.8)	− 0.495	0.621
CD3+ CD4+	24.9 (20.1–33.4)	28.3 (24.8–38.5)	− 1.156	0.248
CD3+ CD8+	23.5 (20.8–29.2)	25.0 (19.5–30.2)	0	1
CD4/CD8	1.3 (0.9–1.5)	1.2 (0.9–1.5)	− 0.083	0.934
CD3-CD19+	26.2 (20.5–37.5)	25.2 (14.0–32.7)	− 0.661	0.509
CD3-CD (16+ 56)+	10.1 (6.7–19.9)	8.3 (4.9–13.5)	− 1.074	0.283
CD19+ CD23+	9.1 (6.2–13.0)	9.2 (4.8–14.1)	− 0.041	0.967

**Table 4:** Comparisons of population with abnormal laboratory data

Laboratory data	MPNP (n = 11)	N-MPNP (n = 20)	P value
WBC > 10 × 10^9^/L	6 (54.55%)	17 (85%)	0.153
N% > 75%	4 (36.36%)	9 (45%)	0.718
ALT > 50U/L	4 (36.36%)	6 (30%)	1
AST > 67U/L	5 (45.45%)	7 (35%)	0.705
LDH > 380U/L	11 (100%)	10 (50%)	0.005
Fib > 4 g/L	7 (63.64%)	15 (75%)	0.683
DD > 550ug/L	10 (90.91%)	19 (95%)	1
PCT > 0.5 ng/mL	8 (72.73%)	14 (70%)	1
ESR > 20 mm/h	9 (81.82%)	15 (75%)	1

### Imaging features

**Fig. 1:**
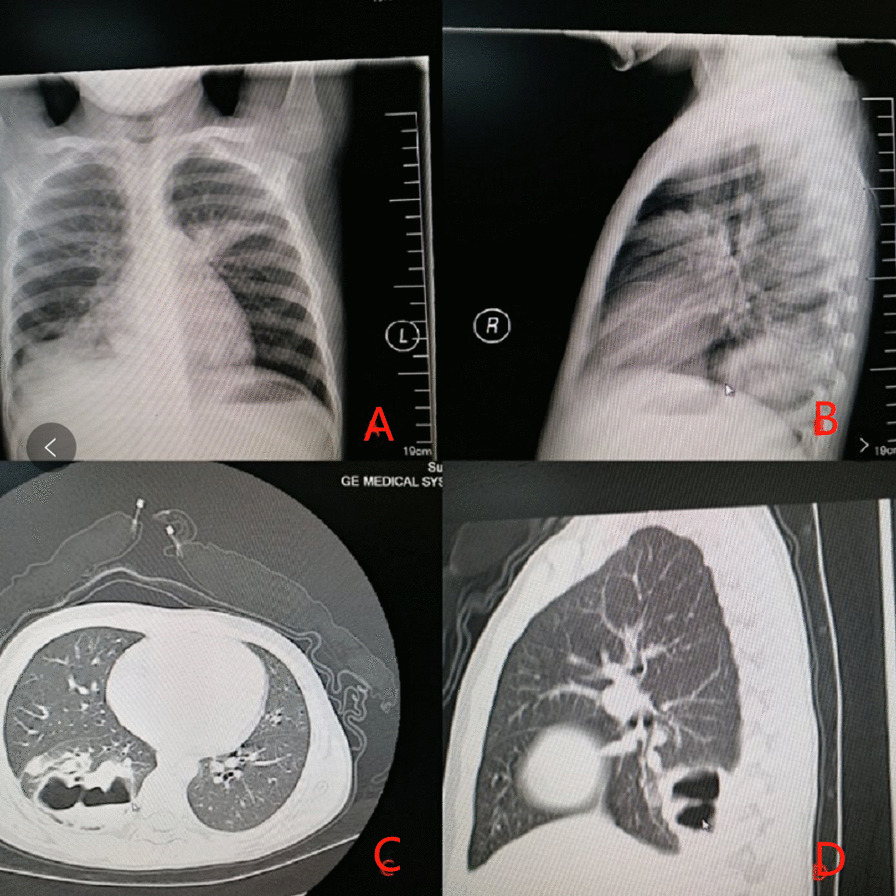
Imaging findings of a 7-year-old male with *Streptococcus pneumoniae* necrotizing pneumonia: **A** and **B**. The disease course was seven days, high-density strip shadow was seen in the right lower lung field, left upper lung field, and left lung postcardiac area, and right diaphragmatic surface and costophrenic angle were blurred. **C** and **D**. The disease course was 40 days, patchy high-density shadow was seen in the right lower lobe, and multiple gas–liquid levels could be seen inside

**Fig. 2:**
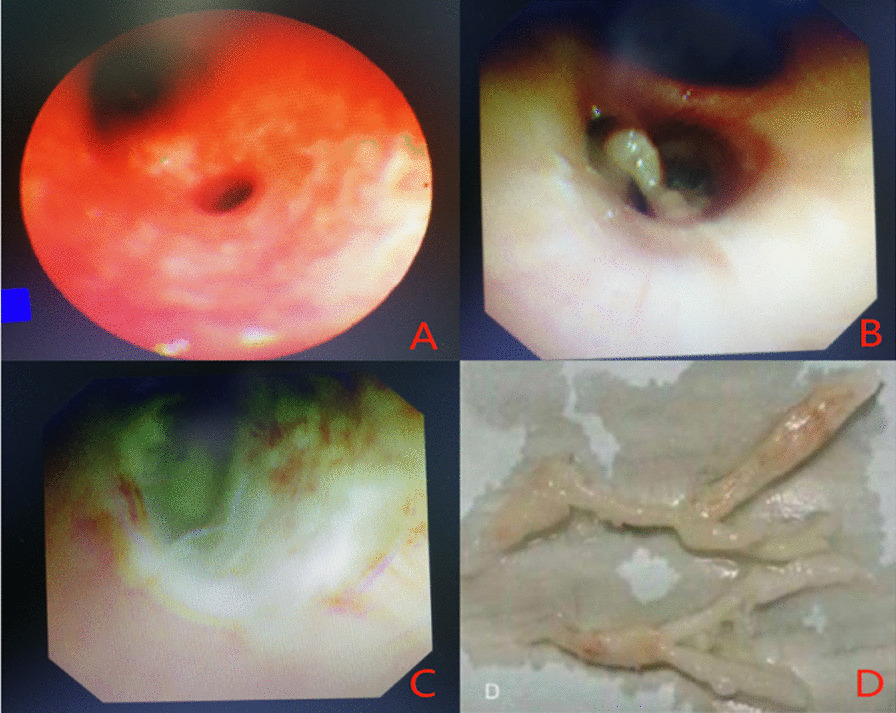
Bronchoscopic manifestations of *Mycoplasma pneumoniae* necrotizing pneumonia in a 7-year-old female (**A** congestion and edema of bronchial mucosa; **B** plastic sputum plug blockage in the lumen; **C** ulcerative erosion surface in the left lower basal branch; **D** plastic sputum embolus clamped out)

All 31 patients showed a large-area lung consolidation in the early stage of chest imaging, and NP was found in chest CT in the later review (Figs. 1, 2). The mean of pulmonary necrosis time (from the onset of the disease to pulmonary necrosis diagnosed by chest CT.) was 18.8 ± 6.3 days of the course of disease in all the enrolled children. The mean of pulmonary necrosis time in the MPNP group was 20.9 ± 6.9 days, which was later than that of the N-MPNP group (16.8 ± 6.1 days; t = 3.101; *P* = 0.004). In addition, the incidence of pleural effusion in the N-MPNP group (19 patients, 95%) was significantly higher than that in the MPNP group (six patients, 54.55%) (*P* = 0.013). According to the early imaging findings, no significant difference was observed in the location of lung parenchymal infiltration and the proportion of empyema, pneumothorax, or atelectasis between the two groups. Only one patient developed a bronchopleural fistula in the N-MPNP group (Table 5).

**Table 5:** Comparison of chest imaging

Chest imaging	MPNP (n = 11)	N-MPNP (n = 20)	P value
Bilateral lobar lesions	4 (36.36%)	4 (20%)	0.518
Right lobe lesion	3 (27.27%)	9 (45%)	
Left lobe lesion	4 (36.36%)	7 (35%)	
Pleural effusion	6 (54.55%)	19 (95%)	0.013
Empyema	0	2 (10%)	0.527
Pneumothorax	1(9.09%)	7 (35%)	0.203
Bronchopleural fistula	0	1 (5%)	1
Atelectasis	2 (18.18%)	1 (5%)	0.281
Necrosis time (d)^a^	20.9 ± 6.9	16.8 ± 6.1	0.004

^a^Necrosis time (d) was in accordance with normal distribution, t = 3.101, P = 0.004

### Treatment, outcome, and follow-up

Treatment methods: all the children were treated with antibiotics (including one or more of β-lactam antibiotics, macrolides, vancomycin, meropenem, or linezolid; one case of *Sparganum mansoni* was treated with praziquantel), and the children with fungal infection were treated with caspofungin at the same time. No significant difference was observed in the duration of use of antibiotics between the two groups (some children’s medication details before hospitalization were unknown). At the same time, most of the children were given different doses of glucocorticoids depending on their condition (methylprednisolone 1–2 mg/kg, intravenous injection, once every 12 h, gradually reduced to oral prednisone sequential treatment, maintained for 5–14 days; some children’s medication details before hospitalization were unknown). A total of 13 children with NP were treated by an intravenous injection of human immunoglobulin (400 mg/kg/d for 3–5 days).

In addition, they were treated by oxygen inhalation, thoracentesis, closed thoracic drainage, invasive respiratory support, extracorporeal membrane oxygenation (ECMO), or bronchoalveolar lavage (BAL). The proportion of oxygen inhalation and mechanical ventilation of the N-MPNP group was 55% and 25%, respectively, however, perhaps because the sample size is too small, there was no significant difference with MPNP group (18.18% and 0, respectively). Two patients were treated with ECMO, one in each group; no statistical significance was observed in the two groups. Moreover, nine patients underwent closed thoracic drainage, three (27.27%) in MPNP, and six (30%) in N-MPNP groups; the proportion was similar (*P* > 0.05). A total of 25 patients among all NP patients were treated by bronchoscopy and BAL because of the poor absorption of lung consolidation by conventional drug therapy. The proportion of BAL was similar between the two groups. However, the proportion of patients receiving multiple (two times or more) BAL was higher in the MPNP group (54.55%) than in the N-MPNP group (30%). Among them, two patients received bronchoscopy lavage at a maximum four times, and the cases of plastic bronchitis were seen only in the MPNP group (three cases; *P* = 0.037). In all patients with NP, only one had pleural fiberboard peeling because of pleural adhesion and pleural collapse (Table 6).

**Table 6:** Comparison of adjuvant therapy

Adjuvant therapy	MPNP (n = 11)	N-MPNP (n = 20)	P value
Bronchoscopy	9 (81.82%)	16 (80%)	1
2 or more times bronchoscopy	6 (54.55%)	6 (30%)	0.255
Phlegm suppository	2 (18.18%)	4 (20%)	1
Plastic bronchitis	3 (27.27%)	0	0.037
Oxygen inhalation	2 (18.18%)	11 (55%)	0.066
Mechanical ventilation	0	3 (25%)	0.535
ECMO	1 (9.09%)	1 (5%)	1
Closed thoracic drainage	3 (27.27%)	6 (30%)	1

### Outcome

Among all the enrolled children, two were discharged (died) after their families gave up the treatment, and these two patients had *Acinetobacter baumannii* infection, one of them had H5N6 infection and been treated with ECMO, and the other one had *Streptococcus pneumoniae* combined with *Acinetobacter baumannii* and filamentous eubacteria infection, accompanied by encephalitis complications. The remaining children had a fever and were discharged after their clinical symptoms improved. The length of stay was 18 (10–22) days in the MPNP group and 23.5 (13.5–47) days in the N-MPNP group and no significant difference was observed between the two groups (Z =  − 1.923, *P* =  − 0.055).

#### Follow-up (Figs. 3, 4)

**Fig. 3:**
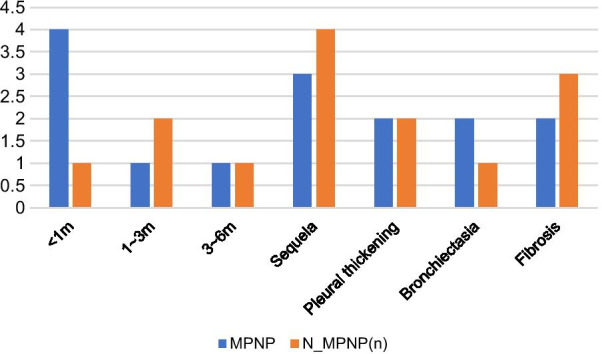
Comparison of imaging recovery time and sequelae

**Fig. 4:**
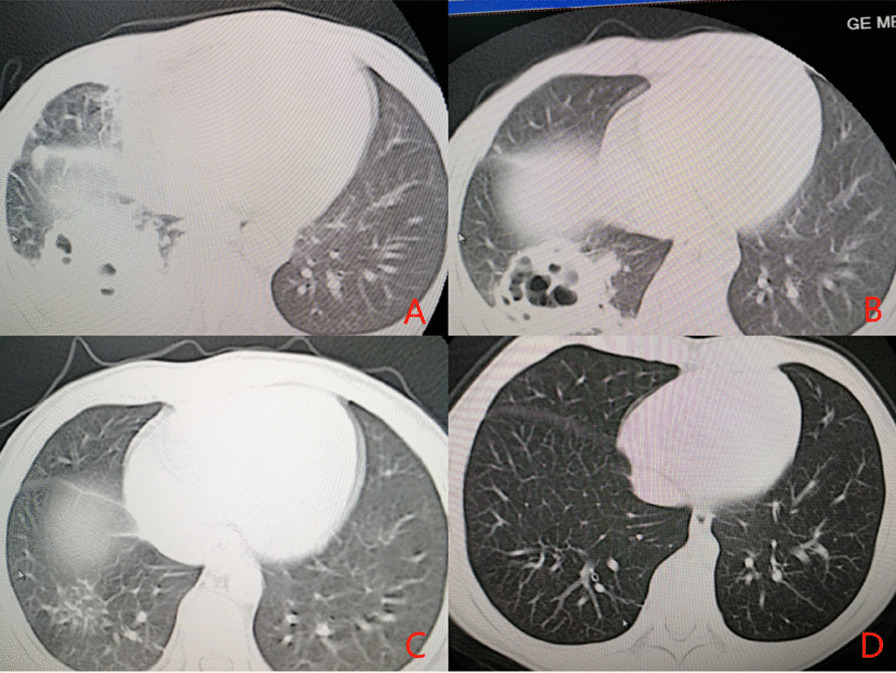
An 8-year-old boy with “intermittent fever for more than one week with chest pain” was admitted.** A** On the eighth day of the disease course, multiple flake and flocculent high-density shadows were seen in the right lung, and the density in the lesions in the lower lobe of the right lung was decreased, and multiple bubble shadows were observed.** B** On the 17th day of the disease course, the flake and flocculent high-density shadows in the right lung were absorbed compared with the former part, and the density in the lesions in the lower lobe of the right lung was decreased, and multiple bubble shadows were observed.** C** On the 51th day of the disease, the flake and flocculent high-density shadows of the original right lung were clearer than the former part.** D** The course of disease was six months. The high-density shadow in the lower lobe of the right lung was observed

Among the 31 patients with NP, two died, 12 (41.38%) were lost to follow-up, and 17 (58.62%) were followed up for a long time. There were nine patients in the MPNP group and eight in the N-MPNP group. The details were shown in Fig. 3. The results showed that most of the children with NP recovered to normal within half a year after discharge. Figure 4 showed a typical case in N-MPNP group recovered well within half a year. The main sequelae were pleural thickening, pulmonary fibrosis, and bronchiectasis. However, no significant difference was observed between the two groups regarding imaging recovery time and the incidence of sequelae. In addition, pneumonectomy was not performed in any child with NP.

## Discussion

The pathological changes in NP include inflammatory infiltration of lung tissue, liquefaction and necrosis of lung parenchyma, and formation of multiple cavities after necrotic tissue clearance [14]. In this study, lung abscess was also included in the NP group for retrospective analysis. In NP, chest CT showed that there were multiple thin-walled cavities or vesicles in the lung consolidation area, which could fuse into a large cavity, some accompanied with liquid level and gas surface. Chest-enhanced CT showed no enhancement at the edge of the cavity, whereas the lung abscess was observed as a single thick wall cavity [12].

At present, the causes and mechanisms of NP are mainly believed to be related to the following two factors: (1) it may be related to various host susceptibility and bacterial virulence factors, and the virus bacteria interaction may also play a role. Some scholars believe that most children with NP have been healthy, which may be an independent risk factor for pulmonary necrosis [15]. (2) Pulmonary capillaries in the necrotic area are blocked by thrombus, which can cause pulmonary parenchymal ischemia and necrosis under the action of inflammatory factors [16].

In recent years, there are several studies on NP in children [16–18]; however, there are few reports on NP caused by different pathogens. Streptococcus pneumoniae and Staphylococcus aureus are the most common pathogens that cause NP in children, and MPNP has been reported mostly in China in the last few years, it may be associated with the increasing number of refractory Mycoplasma Pneumoniae pneumonia and severe Mycoplasma pneumoniae pneumonia cases, decreasing number of severe cases of polyvalent pneumonia vaccination, and the low positive rate of bacterial culture caused by extensive use of antibiotics in the early stage of pneumonia. In this study, 10 cases of N-MPNP were most commonly caused by Streptococcus pneumoniae pneumonia, followed by Staphylococcus aureus pneumonia (four cases), including methicillin-resistant Staphylococcus aureus (two cases) and Acinetobacter baumannii infection (2 cases). In the past, Staphylococcus aureus was considered the typical pathogen of NP. In the past few years, the reports of Streptococcus pneumoniae increased. This study is typically consistent with the reports. Streptococcus pneumoniae is a type of bacteria normally colonized in the human nasopharynx. When the immunity is reduced, Streptococcus pneumoniae causes respiratory tract infection. It is the main pathogen that causes lobar pneumonia. Hsieh YC and other studies have found that the age of more than three years was associated with pulmonary necrosis of Streptococcus pneumoniae pneumonia; however, it is not a confirmed risk factor for NP [15]. In this study, because of the small sample size, NP bacterial pathogen groups and age were not compared. Moreover, two children who gave up treatment died of Acinetobacter baumannii infection, which did not exclude the possibility of infection during invasive ventilator treatment in the hospital. However, one of them was infected with H5N9, which did not exclude the death due to respiratory failure caused by the virulence of the virus; the other patient died of Acinetobacter baumannii combined with Streptococcus pneumoniae and filamentous fungi infection. Considering the possibility of multiple pathogen infections, it is more lethal; however, further prospective studies are needed to confirm this. In addition, the age of children in the MPNP group was significantly older than those in the N-MPNP group, which may be associated with the strong systemic immune response of MP infection in older children.

In terms of clinical characteristics, this retrospective study found that all children with NP had a fever, cough of different degrees, poor spirit, and other manifestations, and the duration of fever lasted for more than 10 days in them; however, no statistical difference was observed between the two groups, and no report exists on the difference of fever time. Moreover, the proportion of patients with dyspnea and the need for oxygen therapy were significantly higher in the N-MPNP group than that in the MPNP group (*P* < 0.05). It may be because of the release of inflammatory factors after bacteria mainly infect the lung parenchyma, resulting in inflammatory infiltration and exudation in the alveolar cavity; thus, reducing alveolar ventilation function [19]. In terms of extrapulmonary complications, severe Steven-Johnson syndrome and central nervous system infection occurred in the N-MPNP group. However, the sample size was extremely small in this study to prove that the extrapulmonary manifestations of the N-MPNP group were severe compared with those of the MPNP group; thus, a prospective study, including large sample size, is needed.

In terms of laboratory examination, LDH levels in the MPNP and N-MPNP groups increased to varying degrees; however, the median LDH level in the MPNP group (805.0 [423.7–1029.5] U/L) was significantly higher than that in the N-MPNP group (414.0 [299.9–540.6] U/L). LDH mainly consists of five isozymes. LDH1 and LDH2 are commonly found in the human heart, kidney, and red blood cells, and LDH3 levels are higher in the lungs [20]. Studies have shown that the body produces excessive immune inflammatory response after MP infection and the release of inflammatory factors causes tissue cell damage; thus, the serum LDH level of patients is significantly increased [21]. Therefore, the serum LDH level in children with NP caused by MP infection was higher than N-MPNP, because of the stronger immune inflammatory response induced by MP infection. In addition, the results showed that the levels of FIB and DD were significantly increased. DD > 1367 ng/mL (OR = 8.501) is a risk factor for NP [16]; however, DD between MPNP and N-MPNP groups was not statistically significant, and no case of pulmonary embolism was observed in this study. In addition, the WBC count was significantly higher in the N-MPNP group than that in the MPNP group. Considering that N-MPNP was mainly caused by bacterial infections, the WBC count was primarily increased. Among inflammatory indexes such as N%, CRP, PCT, and ESR, different degrees of increase were noted, but no significant difference was observed between the two groups. Some studies reported that WBC, N%, CRP, and PCT of children with bacterial NP were significantly higher than that of children with MPNP [22]. However, no significant statistical difference was found in this retrospective study, except for WBC, which may be associated with the long-time use of antibiotics before admission, small sample size, and longer duration of sample collection.

The imaging manifestations of children with NP were generally serious; most of them were complicated with pleural effusion, atelectasis, and pleural thickening. The incidence of pleural effusion in the N-MPNP group was 95%, which was higher than that in the MPNP group (54.55%). A statistical significance was observed between the two groups. The incidence of pleural effusion separation was higher in the N-MPNP group than that in the MPNP group, which may be associated with the activation of coagulation promoting mechanism by the bacterial cell wall.

In this study, the mean time of pulmonary necrosis was 18.8 ± 6.3 days of the disease course, and the mean time of pulmonary necrosis in the MPNP group was longer than that in the N-MPNP group, which may be associated with the older age of children with MPNP, the ignorance of clinicians on NP occurrence, and the delay of lung CT examination. Therefore, in clinical practice, for children with persistent fever, significantly increased WBC, CRP, PCT, LDH and other inflammatory indicators, as well as chest X-ray with large high-density shadow and pleural effusion, it is necessary to be alert to the occurrence of NP and conduct chest CT examination in time.

### Treatment

because of the long duration of fever, high fever peak, and severe illness, all the children in the study were treated with antibacterial drugs (one or more of β-lactam antibiotics, macrolides, meropenem, vancomycin, or linezolid; one patient infected with *Sparganum mansoni* was treated with praziquantel), and the children with fungal infection were given carbamazepine, received longer course of treatment. In this study, children with MPNP were not treated with quinolones or tetracyclines, because in China, the use of such drugs in children is strictly limited for their serious side effects. Also, we didn’t test for resistance gene mutation in the samples which maybe a risk factor of MPNP theoretically. But, in another study published online recently [23], the incidence of macrolides resistance was 95.8%, so we considered whether macrolides resistance is the cause of refractory Mycoplasma pneumonia, so we did further research, but the results were unexpected. We compared children with pneumonia caused by macrolides-resistant Mycoplasma pneumoniae with those caused by sensitive Mycoplasma pneumoniae, and there was no significant abnormality in the imaging examination of lung between the two, and macrolide-resistance may have no relationship with the development of RMPP. The disadvantage is that the blood concentration of macrolide antibiotics was not measured in that study. And there were few articles on the relationship between Mycoplasma pneumoniae resistance and necrotizing Mycoplasma pneumoniae pneumonia too. In the future, further research needs to be carried out in this area. As inflammatory indexes such as WBC, CRP, LDH, and DD increased and pleural effusion was formed, and there may be excessive immune inflammatory reaction mediated by cytokines, most children were given different doses of glucocorticoids at the same time. If an appropriate dose of hormone therapy is given on the basis of anti-infection, it can inhibit the progress of NP or shorten the course of the disease; however, a large sample randomized controlled study is still needed to further verify this.

Intravenous immunoglobulin (IVIG) was given to 13 children with NP. IVIG can neutralize the toxin, reduce the damage to the lung parenchyma, and inhibit the systemic inflammatory response. There is no consensus on the application of glucocorticoid and IVIG in NP. In addition to drug therapy, the clinical application of bronchofibroscopic lavage is highly increased. When children’s airway is blocked by sputum, pulmonary consolidation compresses pulmonary vessels and reduces blood flow; thus, not allowing antibiotics to reach the peak value in the blood, which is required by the pathological site. Alveolar lavage can significantly reduce the number of bacteria and the content of virulence factors; improve the bronchial ventilation function by clearing secretions, and promote the dissolution, absorption, and dissipation of lung consolidation. This study also found that the proportion of children who received BAL two or more times in the MPNP group was significantly higher than those in the N-MPNP group, which may be associated with MP infection, causing atelectasis and requiring bronchoscopy lavage treatment. With the development of fiberoptic bronchoscopy and lavage treatment technology, the operation probability of children with NP was greatly reduced. In this study, only one patient with pleural adhesion thickening and chest collapse underwent pleural fiberboard peeling. In addition, pleural effusion often leads to high fever, shortness of breath, and prolonged course of the disease. To relieve pleural effusion, three children underwent thoracentesis, and nine underwent closed drainage.

Although the onset of children with NP is acute and serious, and the course of the disease is prolonged, compared with NP in adults, most children with NP recover well [3, 6]. All the patients in this study, except for two (both in the N-MPNP group) whose family members gave up treatment, which lead to their death, the rest 29 patients were improved and discharged, and no one underwent pneumonectomy. In the follow-up of 17 children, most of them recovered within half a year. In addition, seven patients had sequelae, four had pleural thickening, three had bronchiectasis, five had pulmonary fibrosis, and several cases had the above-mentioned two or more sequelae, which were consistent with previous reports [24]. Because of the small number of samples in this study and a few follow-up cases, the difference in the proportion of sequelae between the two groups was not evident; therefore, further research is needed.

This study may have the following limitations: (1) this study was a retrospective study, and there may be some bias in case selection; (2) the sample size was small, and the duration of collecting samples was longer, which may have affected the statistical results; and (3) the use of antibiotics, glucocorticoids, and other drugs before admission and the time of chest imaging to find necrosis may have affected the statistical results. Therefore, it is necessary to accumulate more clinical data and further conduct prospective, large-scale, multicenter studies in clinical practice.

## Conclusion

1. MP infection is the most common infection in children with NP in the Suzhou area. There is no gender and age difference between MPNP and N-MPNP, but the bacterial infection was mainly observed in the N-MPNP group.
2. Children in the N-MPNP group have more severe clinical symptoms, were more prone to shortness of breath, had a longer hospital stay, and had earlier imaging manifestations of necrosis, whereas children in the MPNP group were more likely to have plastic bronchitis. The level of WBC and LDH and the nature of pleural effusion can be used to identify MPNP and N-MPNP to some extent.
3. The prognosis of MPNP was better than that of N-MPNP. There were no death cases. Pleural thickening, pulmonary fibrosis, and bronchiectasis were the most common sequelae. Compared with N-MPNP, the recovery time of lung imaging in MPNP was shorter.

## Data Availability

Datasets analyzed in this study can be obtained from the corresponding author upon reasonable request.

## Abbreviations

WBC: White blood cells
N: Neutrophils
CRP: C reactive protein
ALT: Alanine aminotransferase
AST: Aspartate aminotransferase
LDH: Lactate dehydrogenase
Fib: Fibrinogen
DD: D-dimer
PCT: Procalcitonin
ESR: Erythrocyte sedimentation rate
IgA: Immunoglobulin A
IgG: Immunoglobulin G
IgM: Immunoglobulin M
CD: Cluster of differentiation
ECMO: Extracorporeal membrane oxygenation
CT: Computed tomography
MP: *Mycoplasma pneumoniae*
NP: Necrotizing pneumonia
MPNP: *Mycoplasma pneumoniae* necrotizing pneumonia
N-MPNP: Non *Mycoplasma pneumoniae* necrotizing pneumonia

## References

[1] Shi T, McAllister DA, O'Brien KL, Simoes EAF, Madhi SA, Gessner BD, Polack FP, Balsells E, Acacio S, Aguayo C (2017). Global, regional, and national disease burden estimates of acute lower respiratory infections due to respiratory syncytial virus in young children in 2015: a systematic review and modelling study. Lancet.

[2] Griffiths CD, Bilawchuk LM, McDonough JE, Jamieson KC, Elawar F, Cen Y, Duan W, Lin C, Song H, Casanova JL (2020). IGF1R is an entry receptor for respiratory syncytial virus. Nature.

[3] Sawicki GS, Lu FL, Valim C, Cleveland RH, Colin AA (2008). Necrotising pneumonia is an increasingly detected complication of pneumonia in children. Eur Respir J.

[4] Chatha N, Fortin D, Bosma KJ (2014). Management of necrotizing pneumonia and pulmonary gangrene: a case series and review of the literature. Can Respir J.

[5] Kerem E, Bar Ziv Y, Rudenski B, Katz S, Kleid D, Branski D (1994). Bacteremic necrotizing pneumococcal pneumonia in children. Am J Respir Crit Care Med.

[6] Chen KC, Su YT, Lin WL, Chiu KC, Niu CK (2003). Clinical analysis of necrotizing pneumonia in children: three-year experience in a single medical center. Acta Paediatr Taiwan.

[7] Seo H, Cha SI, Shin KM, Lim J, Yoo SS, Lee J, Lee SY, Kim CH, Park JY (2013). Focal necrotizing pneumonia is a distinct entity from lung abscess. Respirology.

[8] Wang Y, Xu D, Li S, Chen Z (2012). *Mycoplasma pneumoniae*-associated necrotizing pneumonitis in children. Pediatr Int.

[9] Chiu CY, Chiang LM, Chen TP (2006). *Mycoplasma pneumoniae* infection complicated by necrotizing pneumonitis with massive pleural effusion. Eur J Pediatr.

[10] Pocket Book of Hospital Care for Children: Guidelines for the Management of Common Childhood Illnesses, 2nd edn. Edited by nd. Geneva: World Health Organization; 2013. pp. 80–91. 24006557

[11] Hansell DM, Bankier AA, MacMahon H, McLoud TC, Muller NL, Remy J (2008). Fleischner Society: glossary of terms for thoracic imaging. Radiology.

[12] Lemaitre C, Angoulvant F, Gabor F, Makhoul J, Bonacorsi S, Naudin J, Alison M, Faye A, Bingen E, Lorrot M (2013). Necrotizing pneumonia in children: report of 41 cases between 2006 and 2011 in a French tertiary care center. Pediatr Infect Dis J.

[13] Veldhoen M, Uyttenhove C, van Snick J, Helmby H, Westendorf A, Buer J, Martin B, Wilhelm C, Stockinger B (2008). Transforming growth factor-beta 'reprograms' the differentiation of T helper 2 cells and promotes an interleukin 9-producing subset. Nat Immunol.

[14] Nicolaou EV, Bartlett AH (2017). Necrotizing pneumonia. Pediatr Ann.

[15] Hsieh YC, Hsueh PR, Lu CY, Lee PI, Lee CY, Huang LM (2004). Clinical manifestations and molecular epidemiology of necrotizing pneumonia and empyema caused by *Streptococcus pneumoniae* in children in Taiwan. Clin Infect Dis.

[16] Zheng B, Zhao J, Cao L (2020). The clinical characteristics and risk factors for necrotizing pneumonia caused by *Mycoplasma pneumoniae* in children. BMC Infect Dis.

[17] Krenke K, Sanocki M, Urbankowska E, Kraj G, Krawiec M, Urbankowski T, Peradzynska J, Kulus M (2015). Necrotizing pneumonia and its complications in children. Adv Exp Med Biol.

[18] Sakamoto N, Tsuchiya K, Hikone M (2018). Community-acquired necrotizing pneumonia with bacteremia caused by *Pseudomonas aeruginosa* in a patient with emphysema: an autopsy case report. Respir Investig.

[19] Spencer DA, Thomas MF (2014). Necrotising pneumonia in children. Paediatr Respir Rev.

[20] Lu A, Wang L, Zhang X, Zhang M (2011). Combined treatment for child refractory *Mycoplasma pneumoniae* pneumonia with ciprofloxacin and glucocorticoid. Pediatr Pulmonol.

[21] Shin JE, Cheon BR, Shim JW, Kim DS, Jung HL, Park MS, Shim JY (2014). Increased risk of refractory *Mycoplasma pneumoniae* pneumonia in children with atopic sensitization and asthma. Korean J Pediatr.

[22] Zhang YY, Dai LM, Zhou YL, Yang DH, Tang LF, Chen ZM (2019). Comparative analysis of clinical characteristics and prognosis between bacterial necrotizing pneumonia and *Mycoplasma pneumoniae* necrotizing pneumonia in children. Zhonghua Er Ke Za Zhi.

[23] Zhang WL, Zhang XX, Gu WJ, Yan YD, Ji W, Zhu CH, Shao XJ, Hao CL (2021). ZR C: Role of macrolides resistance in children with refractory *Mycoplasma pneumoniae* pneumonia. Chin J Appl Clin Pediatrics.

[24] Hsieh YC, Hsiao CH, Tsao PN, Wang JY, Hsueh PR, Chiang BL, Lee WS, Huang LM (2006). Necrotizing pneumococcal pneumonia in children: the role of pulmonary gangrene. Pediatr Pulmonol.

